# Tuberculous pleurisy in an adult patient after cord blood transplantation

**DOI:** 10.1002/jha2.92

**Published:** 2020-09-12

**Authors:** Takaaki Konuma, Susumu Tanoue, Mai Mizusawa, Motohito Okabe, Masamichi Isobe, Seiko Kato, Satoshi Takahashi, Arinobu Tojo

**Affiliations:** ^1^ Department of Hematology/Oncology The Institute of Medical Science The University of Tokyo Tokyo Japan

A 63‐year‐old Japanese woman with advanced myelodysplastic syndrome received unrelated cord blood transplantation (CBT). A computed tomography scan on day 20 showed pleural effusion (PE) on the right lung (Figure 1A) after neutrophil engraftment on day 19. Following the improvement of grade III acute graft‐versus‐host disease (GVHD), which required corticosteroids, PE gradually had improved on day 99 (Figure 1B). However, the right PE progressed for approximately 4 months after CBT (Figure 1C). T‐SPOT.*TB* was indeterminate on day 113, but turned positive on day 120 under corticosteroids. A pleural puncture on day 148 showed a hemorrhagic exudative PE, but Ziehl‐Neelsen stain and the transcription‐reverse transcription concerted reaction were negative. Subsequently, a pleural fluid specimen returned positive for growth of *Mycobacterium tuberculosis*.

**FIGURE 1:**
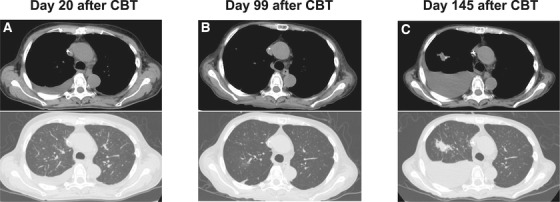
Unenhanced computed tomography scan showing right pleural effusion on day 20 (A), day 99 (B), and day 145 (C) after CBT. Upper: mediastinal condition, lower: lung field condition

The incidence of PE was 9.9% in 618 adult patients after allogeneic transplantation [1]. The most common cause of PE was infectious etiology, followed by volume overload and GVHD, but only one patient had *M. tuberculosis* [1]. Active tuberculosis after CBT is an uncommon infectious complication [2]. The repeated examination by T‐SPOT.*TB* could have contributed to the diagnosis of tuberculous pleurisy in our case. Therefore, tuberculous pleurisy should be considered early after CBT for adult patients with a unilateral PE.

## CONFLICT OF INTEREST

The authors declare no conflict of interest.

## AUTHOR CONTRIBUTIONS

All authors participated in the care of the patient. Takaaki Konuma wrote the first manuscript draft.

## References

[1] Modi D , Jang H , Kim S , Deol A , Ayash L , Bhutani D , et al. Incidence, etiology, and outcome of pleural effusions in allogeneic hematopoietic stem cell transplantation. Am J Hematol. 2016;91:E341‐7. 27238902 10.1002/ajh.24435 PMC6852667

[2] Konuma T , Isobe M , Adachi E , Kato S , Takahashi S , Yotsuyanagi H , et al. Disseminated tuberculosis with cholecystitis in a patient after cord blood transplantation. Intern Med. 2020. doi: 10.2169/internalmedicine.4923-20 PMC7691022 32669496

